# Gastrodiae Rhizoma Ethanol Extract Enhances Pentobarbital-Induced Sleeping Behaviors and Rapid Eye Movement Sleep *via* the Activation of **GABA**_**A**_ ****-ergic Transmission in Rodents

**DOI:** 10.1155/2014/426843

**Published:** 2014-12-30

**Authors:** Jae Joon Choi, Eun-Hye Oh, Mi Kyeong Lee, Youn Bok Chung, Jin Tae Hong, Ki-Wan Oh

**Affiliations:** College of Pharmacy, Chungbuk National University, Cheongju 361-763, Republic of Korea

## Abstract

This research was designed to identify whether Gastrodiae Rhizoma ethanol extract (GREE) enhances pentobarbital-induced sleep *via* *γ*-aminobutyric acid- (GABA-) ergic systems and modulated sleep architectures in animals. GREE (25, 50, and 100 mg/kg, p.o.) inhibited locomotor activity in mice, in a dose-dependent manner. GREE not only prolonged total sleep time, but also reduced sleep latency time in pentobarbital (42 mg/kg)-treated mice. Subhypnotic pentobarbital (28 mg/kg, i.p.) also increased the number of total sleeping animals in concomitant administration of GREE. GREE (100 mg/kg) alone reduced the count of sleep-wake cycles in electroencephalogram. Furthermore, GREE increased total sleep time and rapid eye movement (REM) sleep. From the *in vitro* experiments, GREE increased intracellular chloride level in primary cultured cerebellar granule cells. Protein expressions of glutamine acid decarboxylase (GAD) and GABA_A_ receptors subtypes by western blot were increased. Therefore, our study suggested that GREE enhances pentobarbital-induced sleeping behaviors and increased REM *via* the activation of GABA_A_-ergic transmission in rodents.

## 1. Introduction

Many efforts for the treatment of insomnia have been made by European and oriental scientists from the herbs [1]. Some herbs have been useful for the treatment of mild insomnia [2]. Insomnia is one of the major public health concerns occurring roughly 10–30% in the world populations [3]. The drugs or remedies using treatment of insomnia are targeted to the  *γ*-aminobutyric acid- (GABA_A_-) ergic systems [4]. GABA is the main inhibitory neurotransmitter of the CNS that plays the most important role in the balance of neuronal inhibition [5]. In particular, GABA_A_ receptors have been relevant to control of sleep [6]. Additionally, GABA_A_ receptors complex is contained in Cl^−^ ionophore combined with GABA, barbiturates, benzodiazepines, ethanol, steroids, and picrotoxin binding sites [7]. Primary structures of GABA_A_  receptors compose many kinds of subtypes such as *α* (1–6) subunits, *β* (1–4) subunits, *γ* (1–3) subunits, *δ* (1) subunits, and *ε* (1) subunits [8]. Pharmacological profiles and different behaviors of GABA_A_ receptors agonists depend upon their subtypes [9].

Rhizoma of* Gastrodia elata* Blume (Orchidaceae) has been used traditionally to treat epilepsy, headache, dizziness, and convulsion in oriental countries [10]. The previous study has been focused on treatment of convulsive disorders, such as epilepsy on the central nervous system (CNS) for a long time [11, 12]. GR contains many pharmacologically active compounds, including 4-hydroxybenzaldehyde, vanillin, gastrodin,* p*-hydroxybenzaldehyde, and vanillyl alcohol [13]. In particular, the sedative and hypnotic effects of gastrodin and NHBA [14] have been shown in human and animal models [15].

From these experiments, we focused on whether Gastrodiae Rhizoma ethanol extract (GREE) enhances pentobarbital-induced sleeping behaviors and modulates sleep architecture* via *GABA_A_-ergic systems in rodents.

## 2. Material and Methods

### 2.1. Preparation of Gastrodiae Rhizoma Ethanol Extract

Gastrodiae Rhizoma (GR) was purchased from the traditional market in Cheongju, Republic of Korea. The sample was identified by Professor Mi Kyeong Lee, who works at the Laboratory of Pharmacognosy, Chungbuk National University. The air-dried GR (1 kg) was extracted three times with 50% ethanol at room temperature. The sample was condensed under pressure to give a brown crude extract. After the extract was processed of centrifuge, the filtrate was condensed in vacuum using a rotary evaporator to give dried powder (500 g).

### 2.2. Reagents and Chemicals

Pentobarbital sodium and diazepam were obtained from Hanlim Pharm. Co., Ltd. (Seoul, Republic of Korea) and Samjin Pharm. (Seoul, Republic of Korea), respectively. Muscimol (Tocris Bioscience, Bristol, UK) and dimethyl sulfoxide (Amresco Solon, OH, USA) were purchased, respectively. Fetal bovine serum (FBS), Dulbecco's modified Eagle medium (DMEM), penicillin-streptomycin, and trypsin-EDTA were purchased from GIBCO (Grand Island, NY, USA). The Cl^−^ sensitive fluorescence probe N-(ethoxycarbonylmethyl)-6-methoxyquinolinium bromide (MQAE) and cytosine *β*-d-arabinofuranoside were obtained from Sigma-Aldrich Co. (St. Louis, MO, USA). The specific rabbit polyclonal antibodies against GABA_A_ receptors subunits or GAD_65/67_ and the corresponding conjugated anti-rabbit immunoglobulin G-horseradish peroxidase were purchased from Abcam Inc. (Cambridge, UK). Chemiluminescent HRP substrate was obtained from Millipore Co. (Billerica, MA, USA).

### 2.3. Animals

All animals (4-week-old ICR mice and 8-week-old Sprague-Dawley rats) were purchased from Samtako (Osan, Republic of Korea). The room temperature was maintained at  22 ±2°C and at the relative humidity (50–52%) with a 12 h light/dark cycle in the animal room. Food and water were available* ad libitum*. All rodents were housed in acrylic cages (45 × 60 × 23 cm) and were kept at least 1 week for acclimation time. Experiments were carried out in accordance with the Principle of Laboratory Animal Care (NIH publication number 85-23, revised 1985) and the Animal Care and Use Guidelines of Chungbuk National University, Republic of Korea.

### 2.4. Measurement of Locomotor Activity

Spontaneous locomotor activity was measured automatically with a tilting-type ambulometer (AMB-10, O'Hara, Tokyo, Japan). Each group was processed to adaptation time for 10 min and then measured the movement for 1 h in the activity cage (20 cm in diameter and 18 cm in height) [16]. Groups were administered orally to GREE (25, 50, and 100 mg/kg) and diazepam (2.0 mg/kg) before 30 min and 60 min, respectively. The measurement of locomotor activity was processed automatically for 1 h after sample administration [17].

### 2.5. Measurement of Pentobarbital-Induced Sleeping Behaviors

All mice were fasted for a day. The experiments were carried out between 1:00 and 5:00 pm. Pentobarbital was diluted in 0.9% physiological saline. Muscimol and GREE were suspended in 0.01% DMSO. The groups were administered orally to GREE (25, 50, and 100 mg/kg) and muscimol (0.2 mg/kg) before 1 h and 30 min, respectively, and then pentobarbital (42 mg/kg, i.p.) was injected to all mice (0.1 mL/10 g). The animal that stopped the movement after pentobarbital injection was moved carefully to another cage. The sleep latency was recorded as elapsed time between pentobarbital injection and loss of the reflex. The sleep was recorded as elapsed time between the loss and recovery righting movement. Mice which failed to induce sleep within 15 min after administration of pentobarbital were excluded from the experiments [18, 19].

### 2.6. Implantation of the EEG Telemetric Transmitter in the Rat

Rats were anesthetized by administering pentobarbital (50 mg/kg, i.p.). The animals were then placed on a pad and then secured in the stereotaxic apparatus under aseptic conditions. An incision was made in the part of the abdomen's midline, caudal to the cartilage, and the transmitter (Data Sciences International TA11CTA-F40, MN, USA) was placed in subcutaneously. It was attached to the skin with 3 sutures for stabilization. The periosteum was removed and hemostasis was achieved with sterile cotton. Two holes for positioning the electrodes were drilled in the skull surface (A: 2.0 (Bregma), L: 1.5; P: 7.0 (Bregma), L: 1.5 contralateral). Lines from the transmitter were connected subcutaneously and to the hole that made in the skull. The electrodes lines were anchored to the skull with screws and dental cement. It was fixed by a silk 4–0 suture. For a better recovery all rats were given an antibiotic (i.p.) (5-million-unit potassium penicillin-G injection, Kunwha Co., Republic of Korea).

### 2.7. Data Collection

Following 7 days postsurgical recovery, rats were divided by control group and sample group. GREE (100 mg/kg) was suspended in 0.01% DMSO and administered orally 1 h before the recording. Telemetric recording of cortical EEG and activity were designed with a slight modification as previous reports [20]. The setting of EEG signal was amplified and arranged as 0.5–20.0 Hz in the transmitters. It was additionally set up −0.5/+0.5 volts/unit × 2 and was controlled by a Data Sciences International analog converter and passed to an AD converter (Eagle PC30, USA) saved* via* computer. The signal was digitized at a sampling rate of 128 Hz by an AD converter. Results of recording marked graphically were possible to express* via* the computer. An online fast Fourier transformation (FFT) was performed on the EEG data at 2 sec intervals during data acquisition (256 samples) after a Hanning window treatment. The FFT analysis generated power density values from 0.0 to 20.0 Hz at a resolution of 0.5 Hz. The FFT data were further averaged in the range of 0 to 20 Hz for every 10 sec. The sleep data and FFT results were saved on the hard disk every 10 sec for additional offline analysis. Movement of the animal in relation to the telemetry receiver generated transistor-transistor logic (TTL) pulses that were collected and counted as a measure of activity. Data were recorded between 11:00 am and 5:00 pm EEG. The activity was recorded simultaneously in each rat.

### 2.8. Data Analysis

The states were scored visually from polygraphic recordings stored with SleepSign 2.1 software (KISSEI Comtec Co. Ltd, Matsumoto, Japan). Every 10 sec period was classified as amount of time in wakefulness, nonrapid eye movement (NREM), and rapid eye movement (REM) [21]. The software was classified as EEG signal according to following: wakefulness was identified by a high frequency. Recoding of NREM was focused on the presence of spindles interspersed with slow waves. EEG power during REM is significantly reduced in lower frequency *δ*-wave (0.75–4.0 Hz) and increased in the range of *θ*-wave activity (5.0–9.0 Hz, peak at 7.5 Hz). The time spent (min) in NREM, REM, total sleep time (NREM + REM), and counts of sleep-wake cycle were processed to get 6 h total for each rat. We also calculated the time of each recording spent in each sleep-wake state (wake, NREM, and REM). The absolute EEG power was calculated during wakefulness, NREM, and REM in 0.5 Hz bins from 0.5 to 20 Hz for the entire 6 h reading. The EEG power density was evaluated in 3 selected frequency bands for wakefulness, NREM, and REM (*δ*-wave, *θ*-wave, and *α*-wave (8.0 to 13.0 Hz)). The values were then exported to Microsoft Excel to calculate the amounts of each vigilance state.

### 2.9. Cell Culture

Primary cultures of cerebellar enriched in granule cells were prepared from 8-day-old Sprague-Dawley rats [22, 23]. Cerebellar granule cells were seeded, respectively, in 96-well microplates (1.0 × 10^5^ cells per well) dishes coated with poly-L-lysine (50 *μ*g/mL; Sigma, St. Louis, MO, USA). The cells were resuspended in the following culture media: DMEM, 10% heat-inactivated fetal bovine serum, glutamine (2.0 mM), gentamicin (100 *μ*g/mL), antibiotic-antimycotic solution (10 *μ*g/mL; Sigma), and potassium chloride (25 mM). After seeding the cells, incubated in a humidified atmosphere of 5% CO_2_/95% air at 37°C for 6-7 days. Cytosine arabinofuranoside (final concentration, 10 *μ*M; Sigma) was added after 16 h of culture to inhibit the replication of nonneuronal cells.

### 2.10. Measurement of Intracellular Chloride Influx

The intracellular Cl^−^ concentration ([Cl^−^]i) of cerebellar granule cells of rats was estimated using Cl^−^ sensitive fluorescence probe MQAE according to the method of West and Molloy, with a slight modification [24]. After overnight incubation, the cells were washed three times in the buffer (pH 7.4) which contained 2.4 mM HPO_4_^2−^, 0.6 mM H_2_PO_4_^−^, 10 mM HEPES, 10 mM D-glucose, and 1 mM MgSO_4_. Fluorescence was monitored using excitation and excitation wavelength 320 nm and emission wave length 460 nm, respectively, in a microplate reader (SpectraMax M2e Multi-Mode, PA, USA) as described by previous report [25]. The data were calculated as the fluorescence *F*/*F*_0_ on the basis of the Cl^−^ ratio (*F* is the fluorescence as a function of each sample and *F*_0_ is the fluorescence without Cl^−^ ions).

### 2.11. Western Blotting of Overexpression of GAD and GABA_A_ Receptors Subunits

The proteins were extracted from the primary rat hypothalamic cell cultures. GREE was suspended in 0.01% DMSO and diluted, respectively, in culture medium to final concentrations of 10 and 100 *μ*g/mL. Control samples were treated with solvent alone at the same dilution as that used for drug treatment. The culture medium was completely replaced with fresh medium containing the appropriate drug. After treatment of pentobarbital and GREE, the cells were harvested and treated with cold lysis buffer (25 mM Tris-HCl (pH 7.4), 150 mM NaCl, 1 mM CaCl_2_, 1% Triton X-100, 1 mM PMSF, 10 *μ*L/mL aprotinin, 1 mM NaF, and 2 mM sodium orthovanadate). The extracts were centrifuged at 13,000 ×g at 4°C for 20 min, and the supernatant was taken. Protein concentrations were determined using the Bradford protein assay [26]. The samples were stored at −20°C.

Equal amounts of protein were loaded and electrophoresed on 10% SDS, polyacrylamide gel. Proteins were then transferred to PVDF membranes (Hybond-P, GE Healthcare, Amersham, UK) using a semidry transfer system. The blots were blocked for 1 h at room temperature with 5% (w/v) BSA (applied to all primary antibodies except for glyceraldehyde 3-phosphate dehydrogenase (GAPDH)) and 5.0% (w/v) skim milk (only applied to GAPDH) in tris-buffered saline solution (TBS) containing 0.1% Tween-20. Both specific rabbit polyclonal antibodies against GABA_A_ receptors subunits and rabbit anti-GAD_65/67_ polyclonal antibody at the appropriate dilution in TBST and 5.0% BSA (1 : 2,500 for all the primary antibodies used) were incubated for overnight at 4°C. Blots were washed and incubated with the horseradish peroxidase-conjugated secondary antibody (1 : 3,000 for goat anti-rabbit IgG) in TBST for 4 h at room temperature. The chemiluminescent substrate for the secondary antibody was developed using the ECL detection system (Roche Diagnostics, Mannheim, Germany).

### 2.12. Statistical Analysis

All statistical analyses were calculated by Sigma Stat software (SPSS Inc., Chicago, IL, USA). The results were expressed as mean ± S.E.M. for the number of experiments. Statistical significances were compared between treated group and control group by Holm-Sidak test. Each experiment was repeated at least three times and yielded comparable results. Values with  *P* < 0.05  were considered significant.

## 3. Results

### 3.1. Effects of GREE on Locomotor Activity in Mice

**Figure 1:**
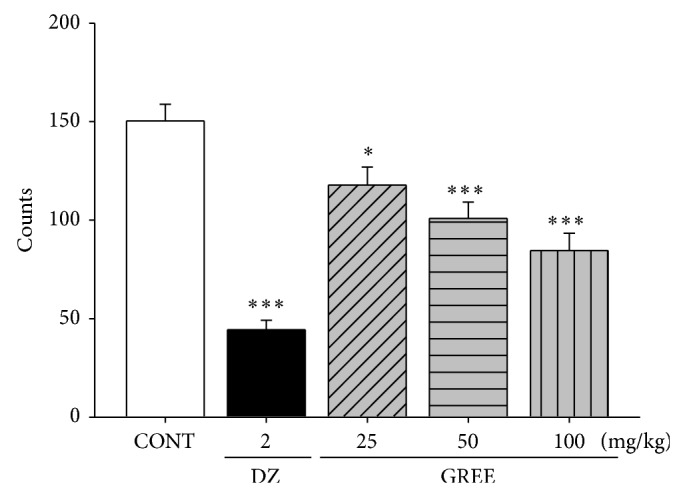
The effects of GREE on locomotor activity test in mice. Ambulation activity was measured for 30 min after oral administration of diazepam and 1 h after the administration of GREE. Each column represents the mean with S.E.M. (*n* = 10). The significance of the effects of the compounds was assessed using analysis of variance (ANOVA) followed by Holm-Sidak test. ^*^ *P* < 0.05, ^***^ *P* < 0.005, compared to the naïve control.

Locomotor activity showed behavior change by diazepam and GREE. By treating diazepam (2.0 mg/kg) and GREE (25, 50, and 100 mg/kg) to mice, locomotor activity was significantly decreased (Figure 1).

### 3.2. Effects of GREE on Pentobarbital-Induced Sleeping Behaviors in Mice

**Figure 2:**
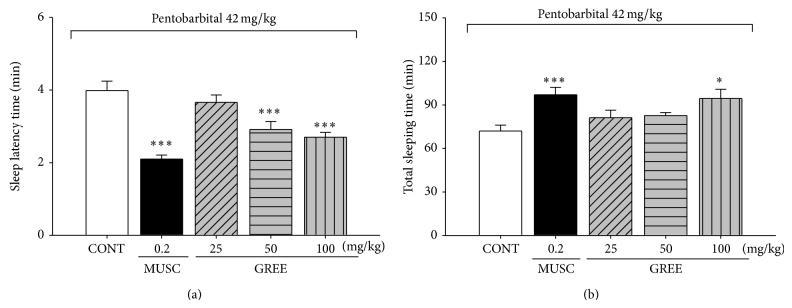
The effects of GREE on onset and duration of sleep in pentobarbital-treated mice. Mice were food deprived for 24 h prior to the experiment. Pentobarbital (42 mg/kg, i.p) was injected to mice following injection of muscimol or GREE. The sleep latency time (a) and total sleeping time (b) were recorded. Each column represents the mean with S.E.M. (*n* = 12–14). The significance of the effects of the compounds was assessed using analysis of variance (ANOVA). Where there was significant variability, the individual values were compared using Holm-Sidak test. ^*^ *P* < 0.05, ^***^ *P* < 0.005, compared with that of the control.

GREE (50 and 100 mg/kg) significantly decreased the sleep latency of sleep. However, 100 mg/kg GREE only increased total sleep duration significantly. Pretreatment of muscimol (0.2 mg/kg, i.p.), a positive control, 30 min before the administration of pentobarbital (42 mg/kg), also showed increase in the sleeping time and a decrease in the sleep latency of sleep (Figure 2).

### 3.3. Effects of GREE on Sleep Onset by Subhypnotic Dosage of Pentobarbital in Mice

GREE reduced the sleep onset time and prolonged the sleep duration induced by subhypnotic dosage of pentobarbital (28 mg/kg, i.p.). Similarly, muscimol group (0.2 mg/kg, i.p.) affected sleeping behavior induced by pentobarbital significantly (Table 1).

### 3.4. Effects of GREE on the Sleep-Wake Cycles

**Figure 3:**
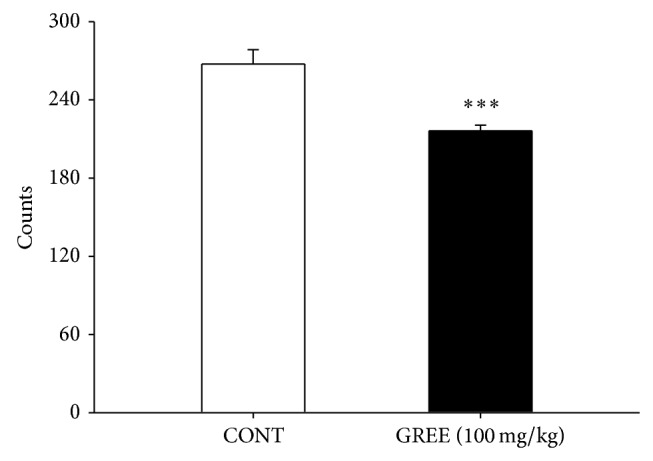
The effects of GREE (100 mg/kg) on counts of sleep-wake cycles in the rats for 6 h. Where there was significant variability, the individual values were calculated as mean with S.E.M. (*n* = 8) and were compared using Holm-Sidak test. ^***^ *P* < 0.005, compared with that of the control.

After GREE (100 mg/kg, p.o.) treatment, sleep-wake cycles were observed in rats during 6 h. The sample group significantly decreased cycle counts compared to the control group (Figure 3).

### 3.5. Effects of GREE on the Sleep Architectures

**Figure 4:**
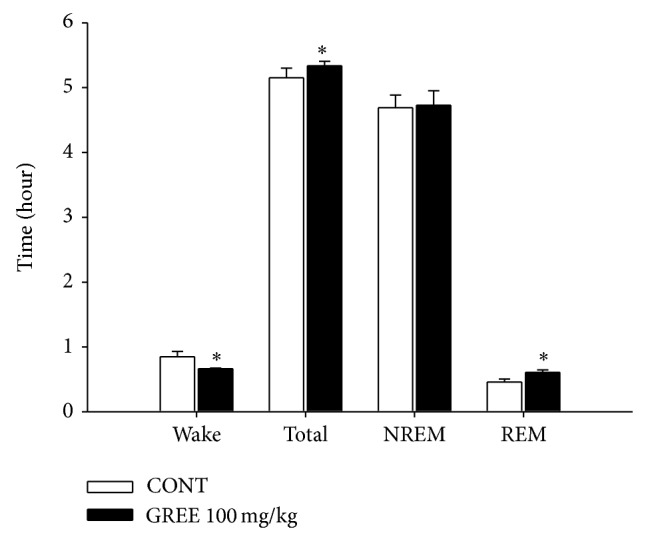
The effects of GREE on rat sleep structure. The data represent the mean with S.E.M. (*n* = 8) of time spent, which separated the wakefulness/sleep (NREM and REM sleep) state. The significance of the effects of the compounds was assessed using analysis of variance (ANOVA). Where there was significant variability, the individual values were compared using Holm-Sidak test. ^*^ *P* < 0.05, compared with that of the naïve control.

100 mg/kg GREE was administered orally to the rat. It significantly increased total sleep and REM but decreased wakefulness. The experiment processed recording per 6 h periods (Figure 4).

### 3.6. Effects of GREE on EEG Power Density of REM and NREM Sleep

**Figure 5:**
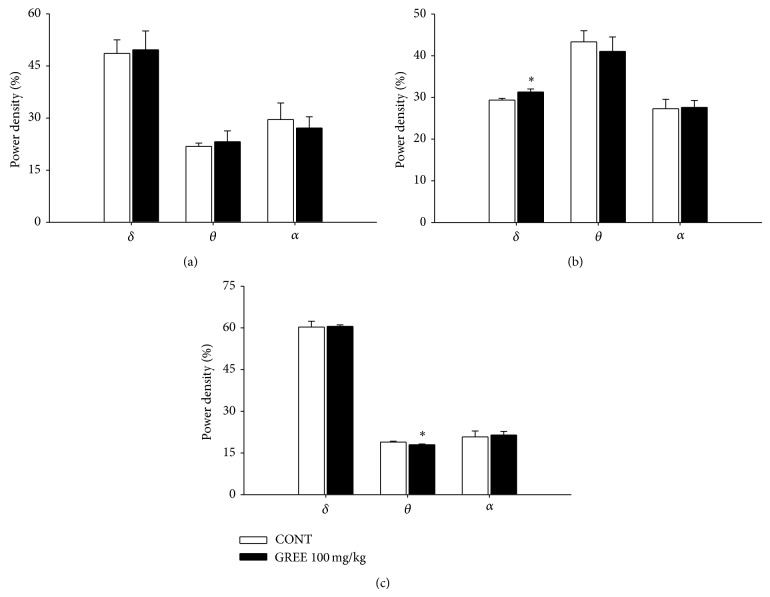
The effects of GREE on EEG power density of wakefulness (a), REM sleep (b), and NREM sleep (c). The power density was departmentalized into *δ*-wave, *θ*-wave, and *α*-wave. Each wave represents the mean with S.E.M. (*n* = 8). The significance of the effects of the compounds was assessed using analysis of variance (ANOVA). Where there was significant variability, the individual values were compared using Holm-Sidak test. ^*^ *P* < 0.05, compared with that of the naive control.

During the measure, it showed a change of wakefulness, REM, and NREM by GREE (100 mg/kg, p.o.) (Figure 5). GREE significantly increased *δ*-wave during the REM power density. However, *θ*-wave power was reduced during NREM power density.

### 3.7. Effects of GREE on Intracellular Cl^−^ Influx in Primary Cultured Cerebellar Granule Cells

**Figure 6:**
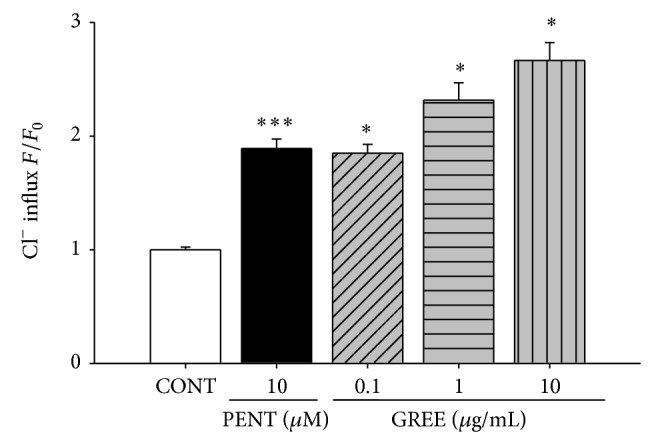
The effects of GREE on chloride influx in primary cultured cerebellar granule cells. After culturing cerebellar granule cells for 8 days, the cells were incubated with MQAE overnight, and then GREE (0.1, 1, and 10 μg/mL) and pentobarbital (PENT 10 μM) were added 1 h prior to measurement. Each column represents the mean with S.E.M. (*n* = 3). The significance of the effects of the compounds was assessed using analysis of variance (ANOVA). Where there was significant variability, the individual values were compared using Holm-Sidak test. ^*^ *P* < 0.05, ^***^ *P* < 0.005, compared with that of the control.

Primary cultured cerebellar neuronal cells enriched in granule cells were calculated. The measured data is presented as the relative fluorescence *F*/*F*_0_, where *F*_0_ is the fluorescence without Cl^−^ and directly proportional to intracellular Cl^−^ concentration. Treatment of granule cells with GREE (0.1, 1, and 10 *μ*g/mL, resp.) produced an increase in intracellular Cl^−^ influx. Interestingly, GREE at all dose concentrations showed significant elevated level of intracellular Cl^−^ influx in this culture model. Pentobarbital at 10 *μ*M concentration as a positive control also increased the influx of Cl^−^ in primary cultured of cerebellar granule cells (Figure 6).

### 3.8. Effects of GREE on the Expression of GAD_65/67_

**Figure 7:**
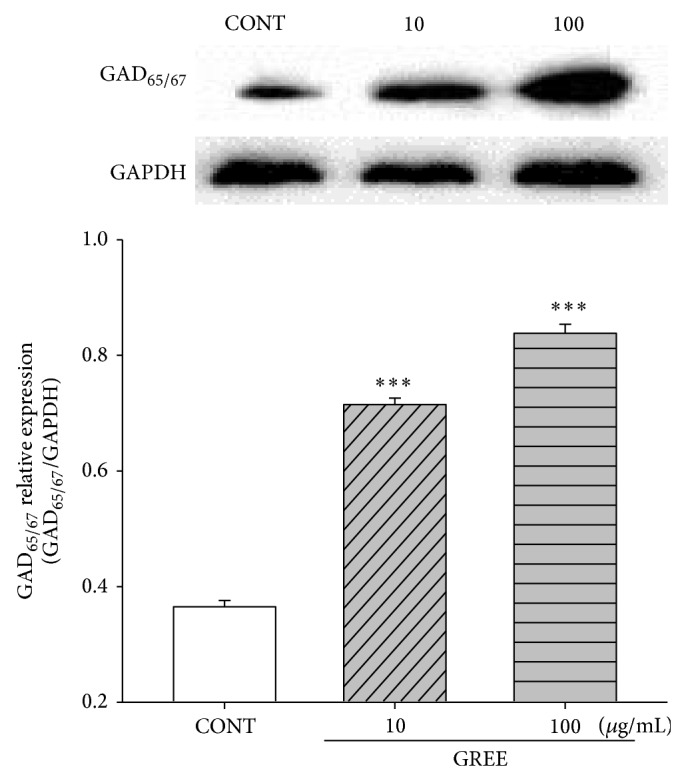
The effects of GREE on the expression of GAD. Immunoblots of lysed hypothalamic neuronal cells which were treated for 1 h following GREE are shown. GAPDH levels were needed for the normalization of the protein expression. Each column represents the mean with S.E.M. (*n* = 3). The significance of the effects of the compounds was assessed using analysis of variance (ANOVA). Where there was significant variability, the individual values were compared using Holm-Sidak test. ^***^ *P* < 0.005, compared with that of the control.

The expression of GAD was examined with lower (10 *μ*g/mL) and higher (100 *μ*g/mL) GREE concentrations. Both concentrations affected the abundance of GAD. High protein content of GAD_65/67_ was significantly detected by western blot for GREE treatment in primary cultured rat hypothalamic cells at both concentrations (Figure 7).

### 3.9. Effects of GREE on the Expression of GABA_A_ Receptors Subunits

**Figure 8:**
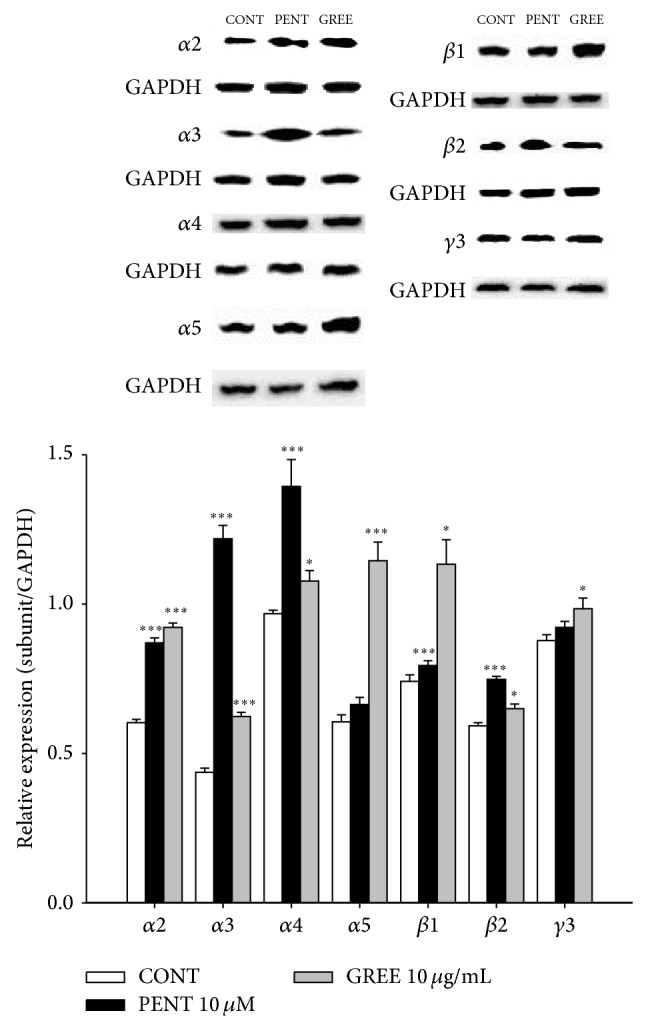
The effects of GREE on expression of GABA_A_ receptors subunits. Immunoblots of lysed hypothalamic neuronal cells which were treated for 1 h following GREE are shown. GAPDH levels were needed for the normalization of the protein expression. Each column represents the mean with S.E.M. (*n* = 3). The significance of the effects of the compounds was assessed using analysis of variance (ANOVA). Where there was significant variability, the individual values were compared using Holm-Sidak test. ^*^ *P* < 0.05, ^***^ *P* < 0.005, compared with that of the control.

GREE (10 *μ*g/mL) and pentobarbital (PENT 10 *μ*M) were treated in primary cultured rat hypothalamic cells for 1 h and were the amounts of *α*-, *β*-, and *γ*-subunits in the GABA_A_ receptors. The 3 types of GABA_A_ receptors showed all overexpressed protein levels in GREE. Pentobarbital was also increased all high protein levels except *α*5 and *γ*3* via* the relative values (Figure 8).

## 4. Discussion

From our* in vivo* experiments, GREE increased pentobarbital-induced sleeping behaviors and REM sleep* via* GABA-ergic systems. From the preliminary experiment, we found that GREE inhibited locomotor activity, showing sedative effects in mice. In addition, GREE not only prolonged total sleep time, but also reduced sleep latency time in pentobarbital (42 mg/kg)-treated mice. Subhypnotic pentobarbital (28 mg/kg) also increased the number of total sleeping animals in concomitant administration of GREE.

GREE has been traditionally used to treat convulsion, epilepsy, and tetanus showing sedative effects. Recent animal and clinical studies indicate that GABA_A_-ergic systems have been thought to be involved in the mechanisms of these inhibitory effects [27]. GREE and its phenolic compounds such as 4-hydroxybenzaldehyde, vanillin, and gastrodin showed anxiolytic-like effects in animal models through activation of GABA_A_-ergic neurons [28]. GREE also showed anticonvulsant effects on pentylenetetrazol-induced seizure [29]. The activation of GABA_A_ receptors shows sedative, hypnotic, and anticonvulsant effects. Therefore, the activation of GABA_A_ receptors induces sleep in the central nervous systems [30]. Therefore, we presume that anticonvulsive, anxiolytic, and sedative/or hypnotic effects can be mediated by GABA_A_-ergic systems. For these reasons, experiments were continued to understand possible mechanisms of GREE which induces sleep.

GABA_A_ receptors complex forms heteromeric GABA-gated Cl^−^ channels assembled from a large family of subunits. GABA_A_ receptors linked chloride channels are opened after binding GABA to give a net inward flux of negative Cl^−^ (outward current), hyperpolarizing the membrane and reducing neuronal firing [31]. To investigate the more precise mechanisms involved in prolongation of pentobarbital-induced sleeping time caused by GREE, Cl^−^ influx was measured in primary cultured cerebellar granule cells after treatment of GREE. GREE increased intracellular Cl^−^ influx. In addition, the activation of GAD is necessary for GABA synthesis [32] and also plays an important role in GABA_A_-ergic systems. GAD activation increases GABA_A_-ergic transmission because GABA is generated from glutamate by the action of GAD. We also confirmed that protein levels of GAD_65/67_ by lower and higher dose of GREE were overexpressed in primary cultured hypothalamic cells.

Furthermore, GABA_A_ receptors possess different binding sites such as GABA, benzodiazepine, and barbiturates. Benzodiazepine agonists such as diazepam enhance the affinity of GABA_A_ for its receptors and hence decrease locomotor activity. GABA_A_ agonist, such as muscimol, directly interacted with the GABA binding site to activate induced sleeping [33]. Similarly, some herbs which interact with GABA_A_-ergic systems induce sedative-hypnotic effect [34, 35]. GABA_A_ receptors subunits were analyzed by overexpressed protein by pentobarbital and GREE in primary cultured rat hypothalamic cells. GREE produced significantly high protein expressions in all expression patterns of 4*α*-subunits (*α*2, *α*3, *α*4, and *α*5), 2*β*-subunits (*β*1 and *β*2), and *γ*-subunit (*γ*3) in GABA_A_ receptors. Pentobarbital also increased high protein levels in all subunits except for *α*5 and *γ*3. From these experiments, molecular targets of GREE are somewhat different from barbiturates, showing different protein overexpressions of GABA_A_ receptors subtypes by western blotting. Further research is necessary to define the molecular targets of GREE.

GABA_A_ receptors agonists reduce wakefulness time and prolong the total REM and NREM sleep. However, these drugs sometimes decrease EEG *δ* activity in NREM sleep [36, 37]. GREE prolonged significantly total sleep time and increased REM sleep except for NREM sleep. The previous research demonstrated that prolongation of REM and NREM sleep as well as special increase of slow waves in NREM plays important roles in the treatment of insomnia and sleep [38]. From our experiments, GREE increased REM sleep and *θ*-waves (slow waves) in REM sleep although it did not increase NREM sleep. It is suggesting that GREE improves the quality of sleep* via *GABA_A_-ergic systems.

## 5. Conclusions

GREE prolonged pentobarbital-induced sleep behavior and EEG in rodents. It also activated GABA_A_ receptors in GABA_A_-ergic systems. GREE increased the Cl^−^ influx in the primary cerebellar cell culture and also increased the protein levels of GAD and GABA_A_ receptors subtypes in the primary cultured hypothalamic cells. GREE showed somnogenic effects and might be useful for the treatment of insomnia* via* the activation of GABA_A_-ergic systems.

## Figures and Tables

**Table 1:** GREE reduced the sleep onset time and prolonged the sleep duration of sleep time induced by sub-hypnotic dosage of pentobarbital (28 mg/kg, i.p.).

Group	Dose (mg/kg)	No. falling asleep/total	Sleep time (min)
Control	0	5/12	20.5 ± 3.8
Muscimol	0.2	11/13^***^	47.2 ± 2.5^***^
GERE	25	9/13	35.6 ± 2.4^*^
	50	11/13^***^	39.6 ± 2.2^***^
	100	13/14^***^	42.3 ± 4.9^*^

Each value represents the mean S.E.M. (*n* = 12–14). ^*^ *P* < 0.05 and ^***^ *P* < 0.005 compared to control.
